# Pembrolizumab plus cisplatin and fluorouracil as induction chemotherapy followed by definitive chemoradiotherapy for patients with cT4 and/or supraclavicular lymph node metastasis (M1Lym) of esophageal squamous cell carcinoma

**DOI:** 10.1007/s00595-024-02867-1

**Published:** 2024-05-20

**Authors:** Nobukazu Hokamura, Takeo Fukagawa, Ryoji Fukushima, Takashi Kiyokawa, Masahiro Horikawa, Yoshimasa Kumata, Yusuke Suzuki, Hironori Midorikawa

**Affiliations:** Department of Surgery, Teikyo University School of Medicine, 2-11-1 Kaga, Itabashi, Tokyo 173-8606 Japan

**Keywords:** Esophageal cancer, Pembrolizumab

## Abstract

Definitive chemoradiotherapy (DCRT) is administered as standard treatment for patients with cT4 and/or M1Lym esophageal squamous cell carcinoma (ESCC); however, its long-term result is inadequate. Although several studies have reported that conversion surgery can improve the survival of these patients, none have identified significantly better long-term survival than that achieved by DCRT. Thus, enhancing DCRT seems important to improve the survival of these patients. A strategy of shrinking tumor volume before DCRT and providing consolidation chemotherapy for systemic control is expected to improve the survival of these patients. Pembrolizumab plus cisplatin and fluorouracil has demonstrated good local control and significant improvement in the survival of patients with advanced esophageal cancer. Based on these results, the following strategy is proposed: This protocol should be applied as induction for these patients; then, DCRT should be provided depending on the initial response; and finally, adjuvant chemotherapy with an immune checkpoint inhibitor should be given to all responders.

## Introduction

Despite recent advances in multidisciplinary treatment, the prognosis of patients with cT4 and/or supraclavicular lymph node metastasis (M1Lym) of esophageal cancer is still unsatisfactory [1]. In the last two decades, radiation therapy techniques have improved and new chemotherapy agents including immune checkpoint inhibitors (ICIs) have been incorporated into esophageal cancer treatment. We reviewed the treatments for cT4 esophageal squamous cell carcinoma (ESCC) and propose a new strategy based on our findings.

## DCRT as a standard treatment for cT4 and/or M1Lym ESCC

Definitive chemoradiotherapy (DCRT) is regarded as standard in the treatment of cT4 and/or M1Lym ESCC [2]; however, studies have shown that the 3-year overall survival (OS) of patients treated with DCRT is still only 10–20% [2]. Moreover, DCRT resulted in late cardiopulmonary adverse events [3], while fistula formation, a formidable complication, developed in 10–30% of patients during or after DCRT [4]. In addition to these severe complications, refractory strictures developed, even in patients with CR [5]. Although DCRT has been used widely to treat cT4 and/or M1Lym ESCC, therapeutic improvement is necessary, with fewer adverse events.

## Surgery for cT4 and/or M1Lym ESCC

Surgical removal is the best way to prevent local recurrence, fatal fistula formation, and refractory strictures. Moreover, the complete removal of cancer cells will promote long-term survival [6]. R0 resection is the most important prognostic factor for patients with cT4 disease and to achieve this, some preoperative treatment is essential. Yokota et al. gave triplet chemotherapy with docetaxel, cisplatin, and fluorouracil (DCF) as induction therapy for cT4 and/or M1Lym ESCC. Although the Kaplan–Meier survival curve of patients with R0 resection was initially superior to that of patients with CR to DCRT, the two survival curves overlapped after 1000 days [7]. Three other retrospective studies also failed to show the significant superiority of conversion surgery to DCRT for long-term survival [8–10]. In addition to promoting survival, a major advantage of DCRT is that it can preserve the esophagus, thereby conserving normal digestive function. After DCRT, dysphagia improved, and oral intake increased [11]. Enhancing the quality and results of DCRT is important for improving the QOL and survival of patients with cT4 and/or M1Lym ESCC.

## Current status of DCRT for cT4 and/or M1Lym ESCC

During the last two decades, new techniques such as computed tomography-based three-dimensional (3D) treatment planning and intensity-modulated radiation therapy (IMRT) have been introduced in radiotherapy, allowing better anatomic visualization and improved target delineation to avoid normal structures [12]. These novel techniques can increase the radiation dose to targets by reducing its delivery to adjacent normal structures, thereby improving its therapeutic effects with fewer adverse events. However, the 3-year survival rate of patients with cT4 and/or M1Lym ESCC who received DCRT with these new techniques was reported as 20–25%, with esophageal fistula developing in more than 30% [13].

To improve the efficacy of DCRT, Higuchi et al. administered DCF, a stronger regimen than cisplatin and fluorouracil, concomitantly with radiotherapy. Although its clinical response and survival improved, there was a high incidence of severe hematological adverse events [14]. Several studies have evaluated induction chemotherapy and/or consolidation chemotherapy combined with DCRT; however, no study has identified significant improvement in the long-term survival of patients with cT4 and/or M1Lym ESCC [15, 16]. Even though irradiation techniques have improved, the chemotherapeutic agents were still fluorouracil, cisplatin-based, and/or taxanes. This seemed one of reasons for the poor prognosis.

## Our proposal

The survival of patients with cT4 and/or M1Lym ESCC was especially poor for those with a large tumor and/or wide lymphatic spread [4, 17]. To improve the therapeutic effect and prolong the survival of patients with cT4 and/or M1Lym ESCC, it seems important to combine chemotherapy that can reduce the size of a tumor effectively before DCRT, and suppress its recurrence afterwards.

ICIs have become a pillar of current cancer treatment, alongside chemotherapy, radiation, and surgery. For esophageal cancer, KEYNOTE-590 was conducted to evaluate pembrolizumab plus cisplatin and fluorouracil as a first-line treatment for unresectable advanced or recurrent esophageal cancer. The OS of the group treated with pembrolizumab was significantly better than that of the placebo group. It also demonstrated a high ORR of 56% among Japanese ESCC patients [18].

Volume reduction is important to improving the therapeutic effect of DCRT and reducing fatal fistula formation in patients with cT4 disease. In KEYNOTE-590, tumor shrinkage greater than 40% occurred within 9 weeks from the beginning of chemotherapy in more than 50% of Japanese ESCC patients (not published). Therefore, the effectiveness of subsequent DCRT will be enhanced with a reduced risk of fatal fistula formation.

**Fig. 1:**
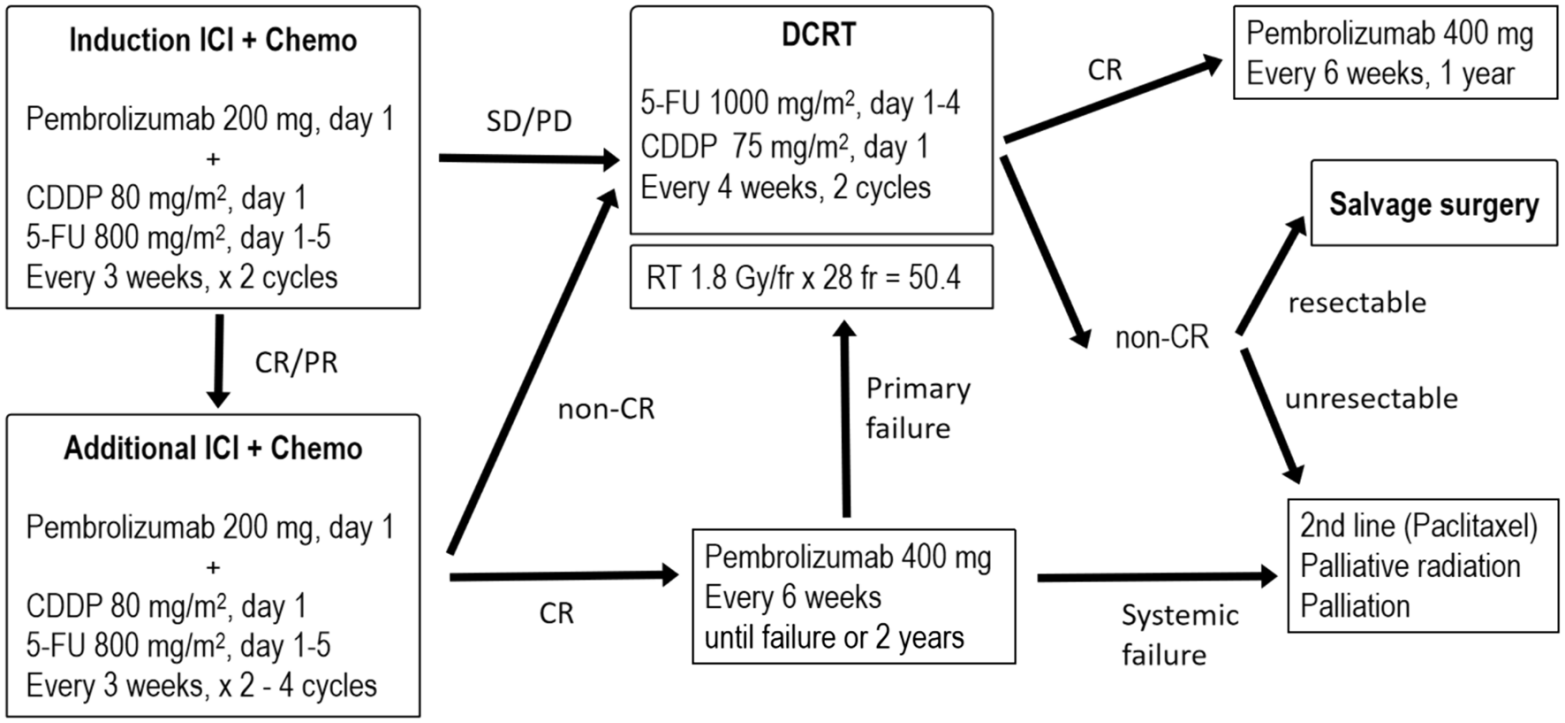
Proposed treatment strategy for patients with cT4 and/or M1Lym esophageal squamous cell carcinoma (ESCC)

Based on this promising antitumor activity, induction chemotherapy combined with pembrolizumab plus cisplatin and fluorouracil is proposed for cT4 and/or M1Lym ESCC, with pembrolizumab 200 mg plus chemotherapy (5-fluorouracil 800 mg/m^2^ on days 1–5 plus cisplatin 80 mg/m^2^ on day 1) once every 3 weeks. After two cycles, the therapeutic effect is evaluated, and patients with CR or partial response (PR) receive two to four additional cycles, depending on adverse events in the initial cycles. Patients with stable disease (SD) or progressive disease (PD) should receive DCRT consisting of radiotherapy (1.8 Gy/fr, 28 fr) with concurrent chemotherapy (5-fluorouracil 1000 mg/m^2^ on days 1–4, 29–32 plus cisplatin 75 mg/m^2^ on days 1 and 29). Patients with CR continue pembrolizumab alone from the seventh cycle because long-term cisplatin use can impair renal function. Patients with CR continue pembrolizumab alone from the seventh cycle because long-term cisplatin can impair renal function. Once renal damage develops, it is difficult to provide DCRT with CF. If local failure develops, DCRT is provided, and if systemic failure occurs, second-line chemotherapy or palliative treatment is provided. If the CR is maintained, this regimen can be continued for 2 years as a protocol, after which they can decide whether to continue. Patients with PR, SD, or PD should undergo DCRT. Patients with CR after DCRT should continue on pembrolizumab alone for 1 year. For patients with non-CR, salvage surgery is performed if the cT4 lesion is relieved. If unresectable, second-line chemotherapy or palliative treatment is provided. Figure 1 details this strategy.

This protocol was developed through a review of treatments for cT4 and/or M1Lym ESCC, but its therapeutic effectiveness has not been evaluated yet. We are now collecting cases treated according to this protocol in our institute, and will report our results.
